# The explanatory models of depression and anxiety in primary care: a qualitative study from India

**DOI:** 10.1186/1756-0500-5-499

**Published:** 2012-09-12

**Authors:** Gracy Andrew, Alex Cohen, Shruti Salgaonkar, Vikram Patel

**Affiliations:** 1 Sangath, Goa, India; 2 London School of Hygiene & Tropical Medicine, London, UK

## Abstract

**Background:**

The biggest barrier to treatment of common mental disorders in primary care settings is low recognition among health care providers. This study attempts to explore the explanatory models of common mental disorders (CMD) with the goal of identifying how they could help in improving the recognition, leading to effective treatment in primary care.

**Results:**

The paper describes findings of a cross sectional qualitative study nested within a large randomized controlled trial (the Manas trial). Semi structured interviews were conducted with 117 primary health care attendees (30 males and 87 females) suffering from CMD. Main findings of the study are that somatic phenomena were by far the most frequent presenting problems; however, psychological phenomena were relatively easily elicited on probing. Somatic phenomena were located within a biopsychosocial framework, and a substantial proportion of informants used the psychological construct of ‘tension’ or ‘worry’ to label their illness, but did not consider themselves as suffering from a ‘mental disorder’. Very few gender differences were observed in the descriptions of symptoms but at the same time the pattern of adverse life events and social difficulties varied across gender.

**Conclusion:**

Our study demonstrates how people present their illness through somatic complaints but clearly link their illness to their psychosocial world. However they do not associate their illness to a ‘mental disorder’ and this is an important phenomenon that needs to be recognized in management of CMD in primary settings. Our study also elicits important gender differences in the experience of CMD.

## Background

Depressive and anxiety disorders, which are collectively referred to as Common Mental Disorders [CMD], are typically encountered in primary health care settings [1,2]. One of the biggest challenges to the effective treatment of the disorders at the primary care setting is the low rate of recognition of CMD by primary health care workers. A leading reason attributed to explain this low recognition is the dominant somatic presentations of CMD [3-5]. ‘Somatization’ has been defined as the ‘presentation of somatic symptoms in place of personal or social problems which includes an avoidance of speaking about emotional distress by focusing on bodily sensations' [6]. Previous authors have observed that somatization is a common phenomenon associated with CMD in non-western settings [7]. This has been interpreted by some as suggesting that there is a denial among these cultures of the psychological origins of their illness experiences [8]. On the other hand a cross-cultural study covering 15 countries found that the somatic presentation of depression did not vary from one country to another [9]. Our research program in Goa, India, has been studying the explanatory models, epidemiology and treatment of CMD for15 years. In previous research, we focused on describing the explanatory models of CMD among women and their association with maternal health and reproductive and sexual health. In these studies, we reported that although women described a number of somatic complaints, there was no 'denial' of their social and emotional contexts [10,11]. Thus, women readily shared emotional experiences consistent with the core psychopathology associated with CMD, described intimate inter-relationships between somatic and mental 'symptoms', and understood both as being caused by social difficulties. In this paper, we build on our previous work by extending our inquiry on the explanatory models of CMD among primary health care attendees of both genders by addressing the following questions:

1) What are the presenting complaints of CMD in primary care settings?

2) What are the psychological phenomena associated with CMD and how are these related to somatic phenomena?

3) What are the causal models for CMD and, in particular, to what extent are psychosocial models used?

4) Are gender differences observed across these dimensions?

## Methods

### Design

Cross-sectional qualitative study nested in the Manas trial [12].

### Setting

The study was conducted in Goa, a state on the west coast of India with a population of 1.4 million. Goa has been a setting for a number of studies on the epidemiology and treatment of CMD by our group [11,13]. The most widely spoken language is Konkani. The study was conducted in 24 primary health care settings from the private and public sectors, spread throughout the state.

### Sample

Participants (N = 117) attending these primary health centres were purposively selected. The eligibility criteria for participation were: being a resident of Goa, being verbally articulate, consenting to participate and scoring above 7 on the General Health Questionnaire-12 (GHQ-12) which indicated a CMD of moderate-severe degree. The GHQ-12 is a widely used screening questionnaire which has been validated for use in primary health care settings in Goa [3]. In this validation study a cut-point of 7/8 was associated with a misclassification rate of 11% and a PPV of 77%.

The treating doctor assessed the patient for any somatic cause for the complaints and treated the respondents accordingly. All patients received treatment for their CMD as per Manas trial protocols [10].

### Interview guide

In-depth interviews were the data collection method. The interview guide was developed through extensive consultations among all the authors and finalized after being piloted with four respondents. The topics elicited in each interview covered the main components of an illness narrative: the experience of the illness, in particular of somatic and psychological phenomena; the history and course of the illness; and the causal explanations for the illness and its relationship to the social worlds of the participants.

### Data collection

Interviewers were provided with participant background information such as age, gender, address and GHQ score. All interviews were conducted in the homes of the participants and were conducted in Konkani, expect for two respondents who were more comfortable in English. All interviews were recorded and interviewers took field notes. The interviews lasted an average of 45 min to an hour.

### Data analyses

The interviewers transcribed the taped interviews, translating non-English interview responses into English. Special attention was given to the local vocabulary for illness and psychological phenomena; such terms and idioms were included in the English transcriptions along with their English translation. Two researchers (one being GA) extensively read the interviews and through an inductive process generated preliminary coding categories which were based on the original research questions. The reliability of the coding system was tested by carrying out blinded, double coding of four interviews and, as a result, a refined consensus coding category was derived. The broad categories were: (a) presenting complaints; (b) psychological experiences; and (c) causal pathways. After coding all the interviews thematic analyses was conducted to elicit common experiences that emerged from the participants narratives. In addition simple frequencies were tallied for the various symptoms that were described. During the entire process, a number of steps were taken to ensure rigor of the data collection and analysis, e.g., interviewers read each others' transcripts with a random number being reviewed by VP (last author).

### Ethical issues

Subjects participated in a written informed consent process at the time they visited the primary care clinics. All respondents were informed that interviews would take place at their own homes or another location of their choice, assured that participation was voluntary, that refusal to participate would have no impact on treatment provided and that no information identifying the individuals would appear in the reports. Ethical approval for the study was obtained from the Sangath IRB and LSHTM as well as the Indian Council for Medical Research.

## Results & discussion

We were able to recruit 30 male and 87 female participants; this gender ratio reflected the patterns of sex differences in primary health care attendees and the greater burden of CMD in women. The mean age for the male participants was 52 years (sd: 16.2) and 47 years (sd: 12.7) for female participants. The mean GHQ score for males was 8.7 (sd: 0.8) and females was 8.9 (sd: 1) which indicated a relatively high score suggesting moderate to severe CMD (Table1).

**Table 1:** Demographic details and score on the GHQ

	**Male**	**Female**
Mean Age	52	46.94
SD age	16.23	12.67
Mean GHQ	8.7	8.85
SD GHQ	0.87	1.006

Reason for consultation (Table2). The most common category of symptoms reported as the reasons for consulting the primary care physician were sleep problems and weakness/tiredness. More than 3 out of 4 respondents complained of tiredness and weakness while problems with sleep were reported by 90% of respondents. The next most common symptoms were the broad category of aches and pains, in particular headache, generalized body ache and pain in the limbs. Autonomic symptoms, in particular giddiness, palpitations and numbness and cramps, were also frequently reported. There was little difference between men and women in the patterns of presenting complaints apart from the observation that higher proportions of women respondents reported most symptom categories. Women also commonly described gynaecological complaints, notably abnormal vaginal discharge and menstrual problems. Typically, respondents described multiple complaints affecting different parts of the body. Notably, psychological complaints were very rarely reported as the reason for consultation.

**Table 2:** Presenting symptoms/reasons for consulting primary care physician reported by people with common mental disorders

	**Categories**	**Male**	**Female**	**Total**
		**n = 30**	**n = 87**	**n = 117**
1.	ACHES & PAINS	22 (73%)	82 (94%)	104 (89%)
1a	Head	11 (36%)	43 (49%)	54
1b	Limbs	8 (26%)	41 (47%)	49
1c	Generalized	6 (20%)	34 (39%)	40
1d	Back	4 (13%)	24 (28%)	28
1e	Chest	5 (17%)	20 (23%)	25
1f	Abdominal	7 (23%)	14 (16%)	21
1 g	Neck	5 (17%)	13 (14%)	18
2	LACK OF SLEEP	30 (100%)	74 (85%)	104 (89%)
3.	AUTONOMIC SYMPTOMS	21 (70%)	76 (87%)	97 (82%)
3a	Giddiness/fainting	10 (33%)	55 (63%)	65
3b	Palpitations	11 (37%)	49 (56%)	60
3c	Numbness/cramps	3 (10%)	30 (34%)	33
3d	“Blood pressure”	5 (17%)	15 (17%)	20
3e	Difficulty breathing	5 (16.7%)	10 (11%)	15
3f	Burning sensations	2 (6.7%)	10 (11%)	12
3g	Trembling	3 (10%)	5 (6%)	8
4.	WEAKNESS & TIREDNESS	19 (63%)	74 (85%)	93 (79%)
5	LACK OF APPETITE	8 (27%)	26 (30%)	34 (29%)
6.	COLD/FEVER	5 (17%)	23 (26%)	28 (23%)
7	GYNECOLOGICAL SYMPTOMS	NA	33 (38%)	NA

"*“The time I had gone to the doctor I was not well I had aches and was feeling giddy (ghuvol ieta). I had this problem of neck earlier but I didn’t give attention to it; now the pain was unbearable so I went to the doctor. I also had cramps in my left hand. Both my hands would get cramps (vath ieta), I would get cramps while travelling on the scooter with my husband” (46 year old female*"

"*“I had joint pain (Gutnemi duktale); my hands and legs were paining and burning. I am taking his treatment. My joint pain, hand and leg pain is reduced but my head becomes hot (takli ujo jalya bashen jata) and my neck muscles are paining. I get headache, head becomes heavy and feels numb (takli jad javn ieta ani muyeta).” (70 year old female)*"

"*“I had pain (like pins) in the stomach (potan kiskis zatale) and then I was unable to do any work. I also have difficulty in breathing (Ubosh ielya bashen jata).” (60 year old male)*"

Psychological phenomena (Table3). The vast majority of participants described cognitive or/and emotional phenomena on probing. Over 90% of respondents reported emotional phenomena and over 80% described cognitive phenomena. The most common emotional phenomena were irritability and anger, followed by lack of interest in day to day activities or their work. About half the respondents described the emotion of sadness or low mood and almost the same proportion described feelings of being "fed up" (*bezar ailo)* with their situation. Over 10% of the respondents talked about being fed up with life and three respondents described active suicidal thoughts. Cognitive phenomena commonly included thinking too much, worrying and forgetfulness and were more frequently described by women than men. On the other hand, the proportions of respondents describing emotional phenomena were similar in both genders. Gender differences were, however, observed in the types of emotional phenomena: men more frequently described nervousness and fears while women were more likely to describe sadness. Feeling “fed up”, an emotion which was commonly described as “bezar ailo”, had various connotations. Respondents referred to feeling a lack of interest in their work, or feeling fed up with their illness or a particular problem or just being fed up with life. Men were more likely to describe feeling fed up or loss of interest in their work while women were more likely to describe feeling fed up with a personal situation such as having to manage the family finances on her own or interpersonal conflicts with her in-laws .

**Table 3:** Psychological phenomena elicited on probing with people with common mental disorders

	**Categories**	**Male**	**Female**	**Total**
		**n = 30**	**n = 87**	**n = 117**
**1**	**EMOTIONAL PHENOMENA**	**27 (90%)**	**81 (93%)**	**108 (92.3%)**
1a	Anger/irritated	21 (70%)	67 (77%)	88 (75%)
1b	Lack of interest in day to day activities or work	14 (67%)	44 (51%)	58 (50%)
1c	Sad	12 (40%)	44 (51%)	56 (48%)
1d	Fed up of ongoing problems	12 (40%)	41 (47%)	53 (45%)
1e	Nervous/scared	9 (30%)	22 (25%)	31 (26%)
1f	Hopeless/fed up of life	3 (14%)	11 (12%)	14 (12%)
2	**COGNITIVE PHENOMENA**	**21 (70%)**	**75 (86%)**	**96 (82%)**
2a	Thinking too much/worrying	18 (60%)	74 (85%)	92 (79%)
2b	Forgetfulness	3 (10%)	21 (24%)	24 (21%)
2c	Poor concentration	2 (7%)	1 (1.1%)	3 (2.5%)

In general, additional probing was needed with male respondents to elicit psychological phenomena as compared with women who were also more easily able to link their psychological experiences with their somatic concerns. The narratives below illustrate how respondents linked somatic and psychological phenomena while describing their illness experience. Thinking too much and/or worrying and feeling one's head getting heavy and/or feeling very tired that everything was an effort were idioms of expressions that were commonly described together by many respondents.

"*I had gone to Dr. B as I had suffocation in my chest (baron ieta) and was unable to breathe properly (shuskar gevpak zaynaslo), couldn’t get sleep. I got tense (maka ‘tension’ zalem) and I got very forgetful. (50 year old female)*"

"*“I used to get irritated (tidak martali) and angry (rag ietalo) on my children. I did not have patience (pasyens ghevpak zainaslen). I used to drink milk to feel better. I was disturbed by negative thoughts (vait vichar), like I do not have anybody’s help or what is the use of life… thinking about all this I used to cry. I was feeling very sad (khup dukhi distalem) and could not stand for long, my body used to tremble (kollkollo ietalo), and I could not hear loud noise. I often told my children that it is due to my health that I cannot hear this loud noise, like the sound of the television or phone ringing. Whenever I tried to work I felt tired (kam kartukuch puro zatalem). Even if I went out for some time I felt tired and wanted to rest” (42 year old female)*"

"*“I have too much worry and my head becomes heavy and I do not get sound sleep. I get very irritated, feel tired and feel that I don’t have any strength. I do not have interest in any work. I feel sadness.” (70 year old female)*"

"*“I was feeling something, I don’t know, I was worried, I was having thoughts of killing my self. I got retired two years back and whatever money I had received after retirement we spent it and it was over so thoughts of how would I manage financially, the household expenses and education of the children kept worrying me. I felt very tired, even going to the market to bring fish was an effort. I felt like sleeping, did not feel like doing anything. I would feel fed up (bezar). I could not concentrate on anything, I would think a lot about how to manage financially. I felt that something was happening to me and I could not control those worries (financial).” (58 year old male)*"

### Causal explanations

#### Labels

When respondents were directly asked to name their health problem about a fifth of respondents were not able to offer a specific label. Half the respondents stated that "tension" and worry (*chinthop*) was causing their health problems, though none referred to this explanation as a 'mental illness'. 15% of respondents cited weakness as the main cause or label of their illness which in turn was attributed to over work or old age. Only 14% of the respondents labelled their problems as a physical illnesses (e.g. diabetes and hypertension) or surgeries. Women were more expressive on how worrying and ‘thinking too much’ (*chod chintha*) about their problems was having an impact on their health. 55% of women attributed tension and worrying to be the primary cause compared to 37% of men although this was the most commonly cited cause of illness for both genders. Women (17%) were also more likely to attribute the cause of their illness to weakness caused by old age or over work compared to men (0%) while men were more likely to associate the cause to other physical illnesses (26%vs 9%).

#### Triggers

When respondents were asked about how their illness began, most traced it to a major life event, e.g., the death of a spouse, an accident or a physical ailment. They would trace these events as far back as 15 years. Women often identified a gynaecological event, e.g., a hysterectomy, as the trigger. Following such a trigger, many respondents described emotionally distressing episodes in their lives, such as a husband's drinking habit or fights with the neighbour or major life events that they associated with contributing to maintaining or worsening their ill- health over time.

"*“I had been operated for hernia nine years back at Margao and since then I have been having health problems. …I have also had diabetes and a few years back my wife died… since then I have been having problems with work, I feel tired and I frequently get cold”* (75 year old male)"

"*“It’s been 5 years now since I got my uterus removed. Since then I have been having these problems of body ache and weakness. I also have asthma, I have had it since I was 12 years old. I am being treated by Dr C for it; he has given me an inhaler pump. Six years back I was taking treatment from Dr J for asthma. Then recently I started getting scared, and my heart would start pounding. I went to doctor C and he said I have become nervous (“nervos jata”) and he gave me tablets to keep my mind cool. If anyone falls sick I get scared and my mouth goes dry” (48 year old female)*"

#### Life events and social difficulties

The respondents described the course of their illness over time interwoven with life events (Table4). In particular, they described ongoing social difficulties, which they linked to their state of ‘tension’ which they attributed as the cause of their illness being maintained over time. Over half the respondents mentioned economic difficulties, e.g., not being able to manage daily expenses, having lost a job, or not being able to work due to a physical illness. Other common sources of 'tension' were interpersonal conflicts (33%) either within the family or with neighbours, and bereavement (29%). 16% of the respondents linked their worry to a family member's drinking problem and 15% described domestic violence. For women, the domestic violence was associated with having a husband who consumed alcohol regularly. In some cases it was the son who was an alcoholic and abused one or both parents. 14% of the respondents described an illness of a family member as their cause for worry, which, in turn were most often linked to financial difficulties.

**Table 4:** Adverse life events and social difficulties determinants reported by people with common mental disorders

	**Male**	**Female**	**Total**
	**n = 30**	**n = 87**	**N = 117**
Financial	14 (47%)	37 (43%)	51 (44%)
Interpersonal conflict	6 (20%)	33 (38%)	39 (33%)
Bereavement	7 (23%)	27 (31%)	34 (29%)
Alcoholism in family member	2 (6%)	17 (19%)	19 (16%)
Domestic violence	2 (6%)	16 (18%)	18 (15%)
Illness of a family member	0	17 (19%)	17 (14%)
Difficulties in managing work	2 (6%)	21 (24%)	23 (20%)
Old age	6 (20%)	11 (13%)	17 (14%)

"*“What can I tell you? It’s because I keep worrying. What can I do? I cannot avoid worrying. Every person thinks about her problem isn’t it? When I keep worrying I get these problems. I have the tension of educating my children and getting them married. Everything is becoming expensive. My husband will be retired in another 3 to 4 months. Since I am a woman I have all these problems. I have to do all the work myself. Nobody is at home to help me. All these symptoms had started eight years back when I had lost my brother” (45 year old female)*"

"*“I have these problems because I think. I get tense and then I become sick. When I am at home she (wife) talks against me. I have problem with my wife. You know why? When I talk to people she doubts me. Because of this for the past one year I am having problem with my wife. This is a big a “tension” to me. I am suffering because of her. I don’t eat food because of tension. This is the only problem” (52 year old male)*"

Marked gender differences emerged in the patterns of social determinants. In general, women attributed their illness to a larger number of social factors compared with men. Around 30% of the women attributed more than three causes while none of the men mentioned more than two causes. Interpersonal conflict, alcoholism or illness in a family member, domestic violence and over-work were more commonly cited by women. On the other hand, old age was more commonly mentioned by men and this was mainly related to not being able to work which, in turn, was causing financial difficulties or the fear of being dependent on others in old age.

Both men and women explicitly linked their somatic and psychological experiences to social difficulties and often described multiple inter-related social difficulties and other health concerns as illustrated by these narratives.

"*“I am sad. I do not like to get out but I have to go and buy the stuff I need. Nobody will get the things. I worry, thinking about how will we manage, my daughter is of marriageable age, should she always look after us? She should be married. If I could do something, I would have looked after my self and my husband. He too is not keeping well. I would have gone out to work but I cannot even manage to do the household work. My son expired eight months back. Now my daughter who is of marriageable age looks after my husband and me. My daughter in-law went to her mother’s place after my son expired. I do the house hold work like washing cleaning, shopping etc. I find it difficult to manage but I have to do it.” (60 year old female)*"

"*“I was getting palpitations (kallzant gop gop zatalem) all throughout the day, I am a driver by profession. I have been having difficulty in driving because of the pain in my neck. I am worried (khub chintalon) about my family. I had taken a loan for the new vehicle I bought and was thinking about the instalments I have to pay, as I have not been going to work and have been at home due to the pain. I have lost all confidence in myself. I often have extreme body ache, get angry easily and am having a lot of tension on how to manage house expenses; I have a family to feed.” (35 year old male)*"

"*“My husband troubles me so I get tension. Always there are fights and no happiness this increases my thinking. My husband does not understand me. He beats me and my daughter. He is doing this since last two years. One day he pushed me on the bed and it hurt me. When he was beating my daughter I caught him so he beat me again. So all this makes me worry and because of this my diabetics increase and I don’t get sleep. I get tension as I go on thinking about that and so it affects my health. This could be why I have also been also feeling tired and weak during these months, My uterus is removed. My sister had told me not to do the operation but when doctor said that it has to be done I did the operation. My two sisters expired so I have that tension also. I am married to my sister’s husband as my sister expired at the time of her delivery. I told my mother that I won’t marry him as he is my brother-in-law. Then I married him against my wish as everybody insisted” (52 year old female)*"

## Discussion

This paper describes the explanatory models of common mental disorders in primary care attendees in Goa, India. We found that somatic phenomena are overwhelmingly the most frequent presenting complaints and, amongst these, weakness/tiredness, sleep problems and aches/pains are dominant. However, psychological phenomena were relatively easily elicited from the majority of patients and cognitive phenomena such as thinking too much and emotional phenomena such as irritability/anger and loss of interest were the most frequent. The majority of respondents used psychological terms to label or describe their illness; 'tension' was by far the most common label. The vast majority of participants attributed their illness to psychosocial factors, rather than physical health factors. We observed very few gender differences apart from the different patterns of psychosocial difficulties.

Common mental disorders such as depression are already the leading neuropsychiatric cause of the burden of disease globally and this burden is projected to increase [14]. Although there are effective treatments for these disorders which can be delivered in primary care settings [12], there are a number of barriers to their implementation, notably the low recognition rates of the disorders in primary care settings. 'Somatization' has been cited as one reason to explain non-detection ([5,8,15] and our study adds to the growing literature examining CMD through a contextual lens, to examine the extent to which 'somatization' occurs within the context of primary care in India viewed through the lens of the patients' explanatory models.

**Figure 1:**
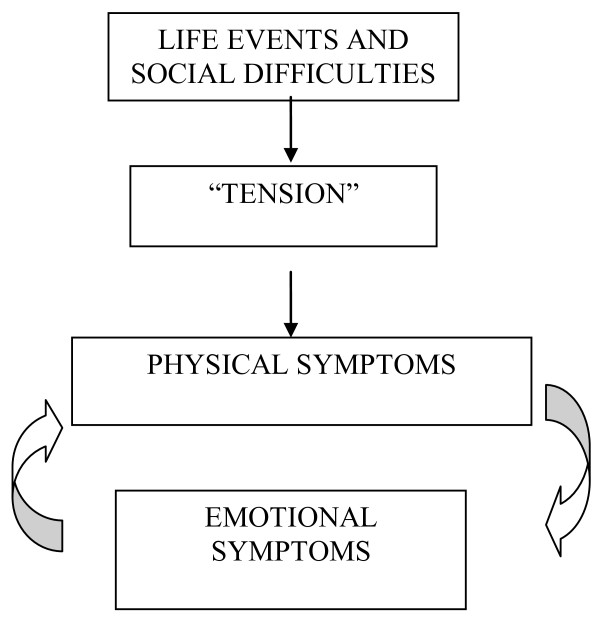
A conceptual model of the casual pathways of CMD.

The findings of our study clearly demonstrate that even though people suffering from CMD predominantly complain of somatic symptoms, these are well recognized hall-mark features of depression and anxiety (for e.g. fatigue and sleep problems). Furthermore, psychological phenomena are easily elicited and the vast majority of patients admit to the psychosocial origins of their illness. Low mood is relatively a less common emotional phenomenon when compared with irritability and anhedonia. Overall, somatic symptoms are clearly located within in a larger bio-psychosocial framework (Figure1); indeed, the 'cardinal features' of CMD as described in contemporary classifications were easily, and commonly, identified by our participants and a substantial proportion used the psychological construct of 'tension' to label their illness. Thus ‘tension” or “worry” appeared to represent the mediating illness category between adverse life events and social difficulties and somatic and mental phenomena. However, none of our subjects considered that they suffered from a 'mental disorder'.

These findings confirm and build on findings that have been reported in previous studies conducted with women in the south Asian region. A study with 39 mothers suffering from post-natal depression, for example, reported that although a high proportion of mothers reported aches and pains, these were most commonly attributed to poor marital relationships and economic difficulties [10]. Another study exploring the relationships between reproductive and mental health in 35 women with CMD reported that women expressed their mental health problems primarily through gynaecological complaints and sleep difficulties but strongly associated them with economic and interpersonal difficulties in their daily lives [11]. In a study from a rural setting, women conceptualized mental health and depressive illness by the presence or absence of ‘pressure’ or ‘worries’, and they considered depressive illness to be directly linked to relational and economic factors in their lives [16].

Similar findings are reported in a study in Chile where patients with mental disorders consulted the primary care doctor for physical complaints but did acknowledge presence of a psychological component to their physical problems [4].

Our study, apart from extending the findings of community based studies on the explanatory models of CMD in South Asia to primary care settings, also adds to the scarce literature on the variations of these models by gender. Our principal observation is that men and women are very similar on symptom patterns, perhaps with the subtle difference that women were more forthcoming during the interviews and described their illness experiences with less probing. This may have implications in the nature of screening and assessment for detection of CMD in primary care. Both men and women described similar determinants linked to economic and interpersonal difficulties; the major differences we observed were that women being more likely to attribute their difficulties to marital conflicts which in some cases are associated with violence and alcohol abuse, and difficulties in managing work, while men were more likely to be concerned about old age, loss of earnings and financial difficulties. These are possibly reflective of the specific social roles that women and men are exposed to in a relatively patriarchal society. It is pertinent to note that the primary determinants cited by our participants in this qualitative study are entirely consistent with the epidemiological findings of the determinants of CMD in the South Asian region which has repeatedly demonstrated the influence of financial difficulties and intimate partner conflict and violence as risk factors [17-19].

While interpreting the findings of this study a number of limitations have to be kept in mind. Firstly, since the patients who participated in the study were identified through a screening tool, there is a small risk of misclassification (small because, as indicated earlier, the tool questionnaire was validated and showed a high PPV at the cut-point used). Secondly, we have used only patient interviews to explore the explanatory models of CMD and these data could have been strengthened by evaluating the patient-PHC doctor encounter and the doctor explanatory models. Finally, the recording of somatic disorder diagnoses was not carried out systematically and thus the possible association of somatic symptoms with somatic disorders cannot be excluded.

## Conclusion

In summary, our findings, and the consistent evidence from other studies of explanatory models in the south Asian region, clearly demonstrates that while somatic symptoms are by far the most common presenting complaints of CMD, 'somatization' as a process is rare. However, even though most patients clearly link their illness to their psychosocial worlds, 'mental disorder' is rarely considered an explanation. In this context, it is notable that studies of what people consider a 'mental disorder' in India have reported that this label is almost exclusively associated with psychotic disorders or intellectual disabilities [20]. There are important implications of these findings to the goal of improving the detection of CMD in primary care settings, most importantly the need to disconnect the labels used to define CMD from 'mental disorder' and affiliate it more explicitly within primary care/family medicine as a stress related disorder with biopsychosocial origins. We believe such a paradigm shift would bring the conceptualization of CMD in line with the epidemiological and ethnographic findings in South Asia and thereby potentially enhance the recognition and management of the disorders and reduce the risk of stigmatization and expectations of specialist care which often accompanies the 'mental disorder' label. Our findings are consistent with those from other non-Western regions of the world, such as sub-Saharan Africa [21,22], suggesting that they may have more global generalizability than those imposed on primary care by contemporary psychiatric classifications and nosology.

## Abbreviations

CMD: Common mental disorders; GHQ-12: General health Questionnaire - 12.

## Competing interests

The authors declare that they have no competing interests.

## Authors’ contributions

GA**,** VP and AC were involved in the design of the study. GA was involved in collection of data, analyses and interpretation of data and preparing first drafts. SS was involved in data analyses and data collection. All authors contributed to and read and approved of the final draft of the manuscript.
